# Ochrobactrum anthropi Causing Fournier's Gangrene: A Report of a Rare Case

**DOI:** 10.7759/cureus.103181

**Published:** 2026-02-07

**Authors:** Tiffani N Kopoian, Cristina Garcia, Alexander Branton

**Affiliations:** 1 Pharmacy, BayCare Health System, St. Petersburg, USA; 2 Infectious Disease, BayCare Health System, St. Petersburg, USA

**Keywords:** antibiotic, bacterial infections, fournier's gangrene, hospital pharmacy, ochrobactrum anthropi

## Abstract

Fournier's gangrene is a form of necrotizing fasciitis that affects the penis, scrotum, or vulva. Novel bacteria for this type of infection include both aerobic and anaerobic bacteria. Rarely, other microorganisms can be the causative agent of the infection. *Ochrobactrum anthropi* is an aerobic, gram-negative, opportunistic infection that often affects orthopedic hardware and has been described as a pathogen within the cornea, as well. This case report describes a patient presenting with Fournier's gangrene in which the causative pathogen was identified via tissue culture as *O. anthropi* that was resistant to most broad-spectrum antibiotics. The patient presented afebrile with leukocytosis, and imaging showed subcutaneous emphysema seen at the soft tissues of the right gluteal cleft and extending along the right base of the penis into the fat of the right inguinal canal, consistent with Fournier's gangrene. Treatment duration and regimen were guided by symptom improvement, existing literature, and the susceptibility report.

## Introduction

Fournier's gangrene is a form of necrotizing fasciitis that affects the penis, scrotum, or vulva [1]. A characteristic finding of Fournier's gangrene is the symptom of disproportionate pain to the outward appearance of the infection [1]. Cases of Fournier's gangrene are typically polymicrobial and result from various aerobic and anaerobic organisms. Some common aerobic organisms are *Escherichia coli*, *Klebsiella *spp., *Proteus *spp., *Staphylococcus *spp., and *Streptococcus *spp. Examples of causative anaerobic organisms include *Bacteroides *spp., *Clostridium *spp., and *Peptostreptococcus *spp. [2]. Rarely, other organisms can be the source of infection. The literature does include case reports of other rare pathogens implicated in the development of Fournier's gangrene. For reference, a case report by Tena et al. describes Fournier's gangrene caused by uncommon bacteria, including *Actinomyces funkei*, *Fusobacterium gonidiaformans*, and *Clostridium hathewayi *[3].

It is difficult to quantify the incidence of unusual bacteria contributing to Fournier's gangrene, as most of them are described only in case reports or case series. Identification of uncommon gram-negative pathogens in necrotizing soft tissue infections can complicate antimicrobial decision-making, particularly given variable resistance patterns and limited organism-specific treatment guidance. Recognizing these uncommon etiologies is clinically important, as early pathogen identification may allow for more targeted antimicrobial therapy concurrent with surgical management.

*Ochrobactrum anthropi* is an aerobic, oxidase-positive, urease-positive, gram-negative, motile, non-lactose-fermenting bacillus belonging to the *Brucellaceae *family and isolated from *Leguminosae *nodules [4,5]. This organism is found ubiquitously in nature that affects both immunocompetent and immunocompromised individuals [2]. A literature review performed by Vaidya et al. described 15 cases of *O. anthropi *infections in immunocompetent hosts, with cases attributed to trauma, endophthalmitis, osteomyelitis, and foreign bodies such as prosthetic valves [6].

Most isolates of *O. anthropi *are commonly resistant to chloramphenicol and beta-lactams, with the exception of carbapenems [2,7]. This is due to its production of AmpC beta-lactamase OCH-1 [4]. Studies have shown susceptibility to gentamicin, fluoroquinolones, sulfamethoxazole/trimethoprim, and colistin. Similar case reports describe varying lengths of antimicrobial therapy, typically ranging from two to six weeks. However, evidence from other studies suggests that a shorter treatment duration (approximately two weeks) may be as effective as longer courses of therapy, with complete recovery [8-10]. At the time of this publication, there have been no reported cases of *O. anthropi *as the isolated agent in a Fournier's gangrene infection, as evidenced by a structured literature search of PubMed from database inception through February 2026 using the search terms "Ochrobactrum anthropi" and "Fournier's gangrene". *O. anthropi *cases affecting the cornea and orthopedic hardware have been reported in the literature [9,11]. An article by Brivet et al. reports on the first case of necrotizing fasciitis associated with *O. anthropi *bacteremia [12].

This case report highlights Fournier's gangrene caused by *O. anthropi*, a rarely reported pathogen in necrotizing soft tissue infections, and highlights the clinical implications of identifying uncommon organisms in severe soft tissue infections.

## Case presentation

A 64-year-old male patient was brought into the emergency department for two ground-level falls with a subsequent traumatic subdural hematoma. The patient's past medical history included traumatic brain injury, atrial fibrillation, hypertension, hyperlipidemia, cervical cord compression with myelopathy, and schizophrenia. Relevant allergies included penicillins. The patient's presentation was significant for abdominal pain, dark emesis, Fournier's gangrene, and altered mental status. The patient was afebrile upon arrival with leukocytosis and critically low hemoglobin. Serum creatinine was elevated. Laboratory work-up is summarized in Table 1.

**Table 1:** Patient baseline laboratory work-up

Test	Results	Reference range
White blood cell count	16.6 th/µL	4.5-11 th/µL
Hemoglobin	5.1 g/dL	14-18 g/dL
Platelets	165 th/µL	140-450 th/µL
Serum creatinine	3.3 mg/dL	0.6-1.3 mg/dL
Creatinine clearance	21 mL/min	70-120 mL/min
Procalcitonin	9.76 ng/mL	0-0.24 ng/mL
Aspartate aminotransferase	67 U/L	5-35 U/L
Alanine aminotransferase	30 IU/L	0-55 IU/L

**Figure 1:**
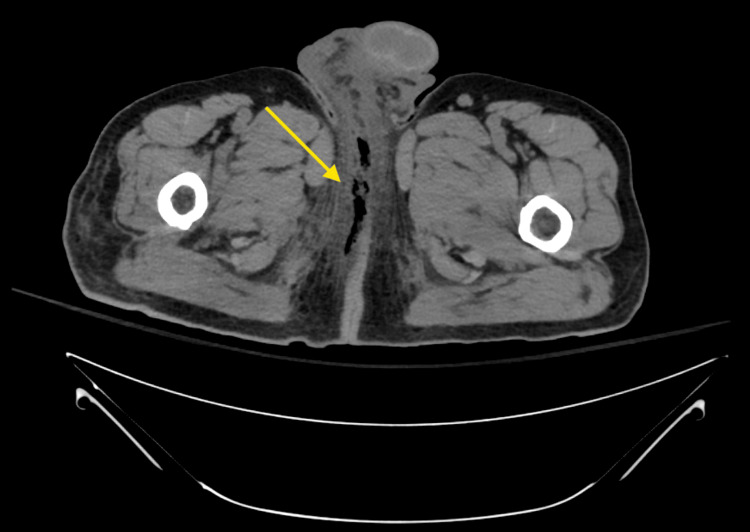
Computed tomography of the abdomen and pelvis

**Figure 2:**
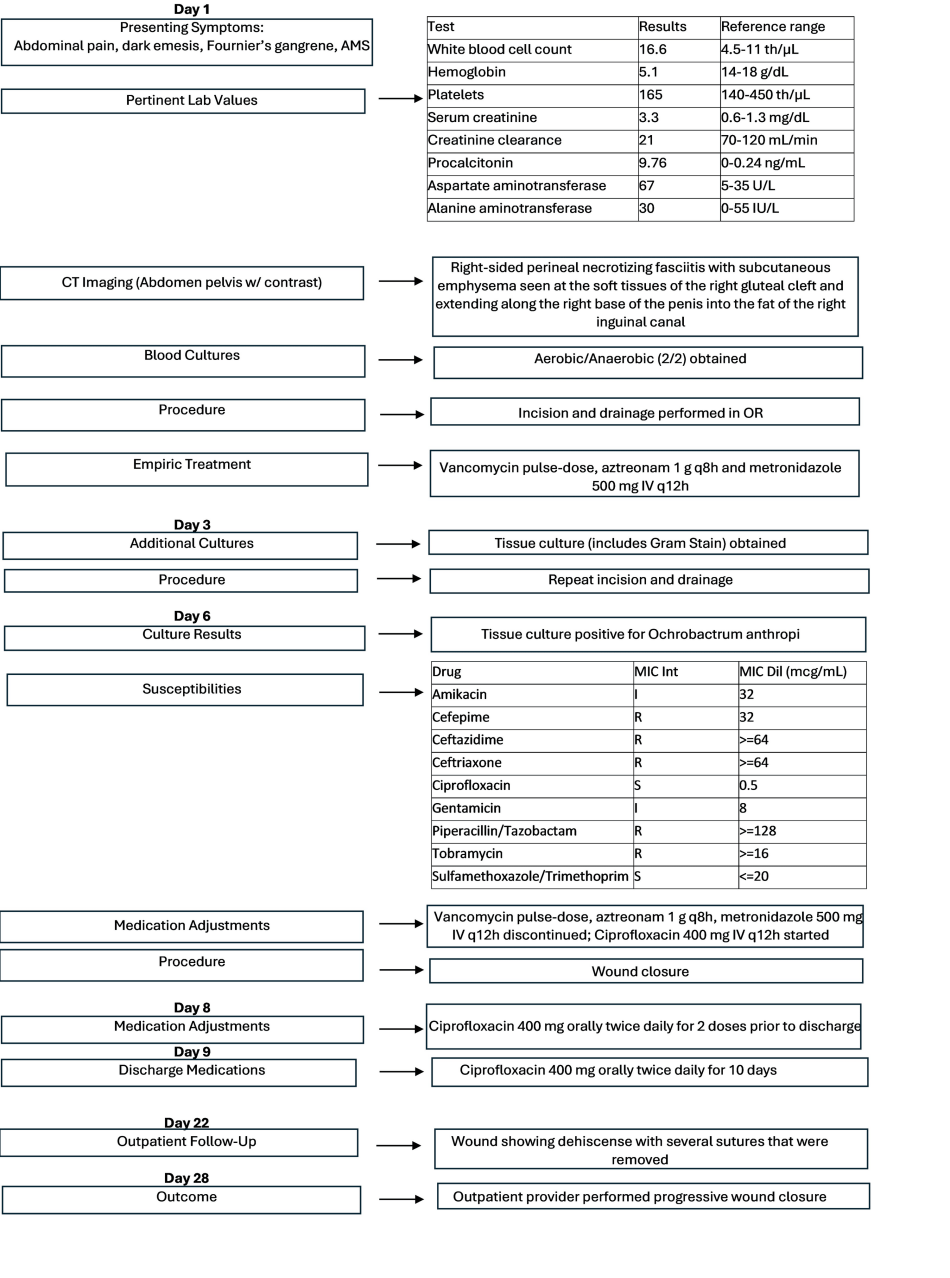
Timeline of events

Computed tomography (CT) of the abdomen and pelvis revealed right-sided perineal necrotizing fasciitis (Fournier's gangrene) with subcutaneous emphysema seen at the soft tissues of the right gluteal cleft and extending along the right base of the penis into the fat of the right inguinal canal, as shown in Figure 1. An infectious disease consult was placed. The deep tissue culture collected from the patient's genital wound revealed scant growth of gram-negative rods, later identified as *O. anthropi*. This was the only organism isolated from the tissue culture. Susceptibility testing was performed using the VITEK® 2 automated system, which utilizes an automated broth microdilution method. Minimum inhibitory concentrations (MICs) were interpreted according to Clinical and Laboratory Standards (CLSI) M100 criteria current at the time of testing [13]. The susceptibility testing revealed that this *O. anthropi *isolate was resistant to cefepime, ceftazidime, ceftriaxone, piperacillin/tazobactam, and tobramycin. The isolate was intermediate to amikacin and gentamicin and showed susceptibility to ciprofloxacin and trimethoprim/sulfamethoxazole. Susceptibilities are shown in Table 2. The patient underwent incision and drainage of the infection site and was initially treated vancomycin pulse-dose, aztreonam 1 g every eight hours and metronidazole 500 mg IV every 12 hours, upon admission, prior to susceptibilities. Antimicrobial therapy was modified within four hours of isolate identification based on the susceptibility report to ciprofloxacin 400 mg IV twice daily for three days before transitioning to oral ciprofloxacin 500 mg twice daily prior to discharge. The patient received two doses of oral ciprofloxacin prior to discharge. Oral ciprofloxacin 500 mg twice daily was continued while outpatient for 10 days. On day 22, the patient was seen for follow-up at an outpatient facility, with the wound beginning to show dehiscence with several sutures that were removed. The outpatient provider recommended progressive wound closure, and this was performed on day 28. A timeline of events is outlined in Figure 2.

**Table 2:** Bacteria susceptibilities MIC Int: minimum inhibitory concentration interpretation; MIC Dil: minimum inhibitory concentration dilution method; I: intermediate; R: resistant; S: susceptible; mcg/mL: micrograms per milliliter

Drug	MIC Int	MIC Dil (mcg/mL)
Amikacin	I	32
Cefepime	R	32
Ceftazidime	R	≥64
Ceftriaxone	R	≥64
Ciprofloxacin	S	0.5
Gentamicin	I	8
Piperacillin/tazobactam	R	≥128
Tobramycin	R	≥16
Sulfamethoxazole/trimethoprim	S	*≤*20

## Discussion

Fournier's gangrene typically occurs from an infection that originates in the perianal or retroperitoneal regions that has spread to the genitalia. Other causes include urinary tract infections or trauma to the genital area [1]. Presentation can include itching, tenderness, edema, and redness of the skin early in the course of the disease and can escalate to systemic symptoms (fever, chills, nausea, etc.). Pain that appears excessive relative to the outward appearance of the infection is common. Fournier's gangrene can be preceded by an event that disrupts the skin's integrity [2].

Per recommendations from the Infectious Diseases Society of America (IDSA), surgical intervention is the mainstay of treatment when Fournier's gangrene is confirmed or suspected. Antimicrobial treatment of Fournier's gangrene consists of broad-spectrum antibiotics of different classes due to the possibility of polymicrobial etiology, with common organisms including but not limited to group A *Streptococcus*, *Staphylococcus aureus*, *Escherichia coli*, and *Pseudomonas aeruginosa *[2]. Empiric antibiotics should include vancomycin, linezolid, or daptomycin plus piperacillin-tazobactam or carbapenem, ceftriaxone and metronidazole, and fluoroquinolone plus metronidazole [1]. The IDSA 2014 Guidelines for the Diagnosis and Management of Skin and Soft Tissue Infections suggest the most effective method for bacteriologic diagnosis is by culture and gram stain of the deep tissue to prevent misleading results that could occur from a superficial wound [14]. Initial empiric antimicrobial therapy was selected in accordance with IDSA guidelines for necrotizing soft tissue infections, which recommend broad-spectrum coverage of gram-positive, gram-negative, and anaerobic organisms. Following identification and susceptibility results, antimicrobial therapy was narrowed to target *O. anthropi*, representing deviation from standard polymicrobial coverage in favor of organism-directed therapy.

*O. anthropi *was described by Holmes et al. in 1988 [15]. The genus was named after the Greek word "Ochros", meaning yellow color [4]. The name "anthropi" was given after it was discovered to thrive in contaminated biologic products, hospital environments, intravascular cannulas, indwelling catheters, and bodily fluids [9].

*O. anthropi *rarely causes human infections due to its low virulence. Studies have identified a limited number of virulence factors in a majority of *Ochrobactrum *isolates. In a study by Yagel et al., all analyzed genomes of *Ochrobactrum *were found to have lipid A biosynthesis genes [16]. Other virulence-associated genes include fatty acid biosynthesis (fabZ (acyl carrier protein)), carbohydrate metabolism (pgm (phosphoglucomutase-1) and cgs (glucan synthesis proteins)), cell wall biosynthesis (wbpL (glucosyltransferase protein)), and biofilm formation (ricA (regulator proteins)) [4].

Recent studies describe *O. anthropi *as an emerging pathogen, affecting immunocompetent hosts and more commonly seen in orthopedic infections [9,6]. This is particularly due to *O. anthropi *adhering to foreign bodies like internal hardware [9]. Risk factors for the development of an *O. anthropi *infection include indwelling medical devices, prior antibiotic therapy, a recent surgical procedure with allografts, co-occurring bacterial infections, and a compromised immune system [5].

## Conclusions

Broad-spectrum antibiotics recommended by the IDSA guidelines should be initiated immediately, prior to culture results, and provide coverage for the common organisms listed above. At the time of this publication, no studies have been published identifying *O. anthropi *as a causative agent of Fournier's gangrene. This case report highlights *O. anthropi *and its ability to cause Fournier's gangrene in immunocompetent adults. Treatment options were limited, and this isolate of *Ochrobactrum *was particularly resistant to most broad-spectrum antibiotics, which did differ from previously published susceptibility profiles of *O. anthropi*. Treatment duration was guided by clinical response, resolution of systemic signs of infection, and surgical source control, as no standardized treatment duration exists for *O. anthropi *soft tissue infections. It did align with the durations documented in previous case reports describing *O. anthropi *infections. Limitations of this report include its single-patient design, which limits generalizability. Findings should be interpreted within the context of the individual clinical scenario. A Fournier's gangrene infection should be considered if perineal, scrotal, or penile swelling and pain disproportionate to physical findings are observed. Once an infected wound is identified, it is imperative to perform surgical debridement of the site and isolate the organism to obtain appropriate susceptibilities for targeted antimicrobial therapy. This guidance was followed while treating this patient and did contribute to the desired outcome of complete recovery.
